# Vertical Marginal Discrepancy of a Monolithic Zirconia Crown with Different Cement Spaces

**DOI:** 10.1155/2023/6698453

**Published:** 2023-12-07

**Authors:** Turki Alkhallagi, Meshari Alzahrani, Majed Ali Alkathiri, Ghadeer I. Basunbul, Amin Marghalani

**Affiliations:** ^1^Oral and Maxillofacial Prosthodontics Department, Faculty of Dentistry, King Abdulaziz University, Jeddah, Saudi Arabia; ^2^Department of Oral and Maxillofacial Surgery, College of Dental Medicine, Umm Al-Qura University, P.O. Box 715, Makkah, Saudi Arabia

## Abstract

The long-term clinical success of indirect restorations highly depends on their marginal integrity. The cement space is an element that might affect the marginal integrity, but it can be altered during the configuring of the computer-assisted designing/computer-aided manufacturing (CAD–CAM) restoration. However, there is controversy in the literature regarding the effect of the cement space on the precision of zirconia crown marginal adaptation. The aim of this study was to measure the vertical marginal discrepancies between different cement thickness settings for CAD–CAM monolithic zirconia restorations. *Material and Methods*. An artificial mandibular right molar tooth mounted on a typodont was prepared for a zirconia crown using the standard method. The study sample consisted of 30 zirconia crowns (Zenostar Zr Translucent Zirconia, Weiland Dental, Germany) milled using an (iMes-iCore) milling machine. Each group of 10 crowns was designed with 30–50 and 70 *μ*m spacer thicknesses. The vertical marginal adaptation at the center of the four different planes (mesial, distal, buccal, and palatal) was measured under a microscope at 40x magnification. A one-way analysis of variance test was used for statistical analysis. *Results*. The mean of Group 30 was 27.45; of Group 50 was 22.22; and of Group 70 was 22.90. There was no statistically significant difference between the groups (*p* ≥ 0.5). *Conclusions*. The increase in the cement space up to 70 *μ*m did not influence the vertical marginal adaptation of the monolithic zirconia crowns.

## 1. Introduction

Porcelain fused to metal (PFM) fixed dental prostheses used to be the gold standard for the restoration of damaged or missing teeth to reestablish their function and esthetics [1]. The introduction of highly aesthetic ceramic restorations has decreased the use of PFM restorations. Several types of aesthetic ceramics are used for anterior and posterior fixed dental prostheses, including zirconia-based ceramics [2, 3]. Zirconia is a high-strength, metal-free, polycrystalline ceramic with no glass content at all [4]. Computer-assisted designing/computer-aided manufacturing (CAD–CAM) technology has increased the use of zirconia ceramics, which has decreased their fabrication costs while improving productivity and saving laboratory time [5, 6].

Traditional zirconia ceramic restorations have an opaque white appearance, which compromises their aesthetics and makes it essential to veneer them with feldspathic porcelain [7]. However, numerous reports have shown that veneered zirconia crowns have a higher rate of chipping and cracking at the core–veneer junction than other crown restorations [8–11]. Individual crowns demonstrated chipping rates ranging from 2% to 9% over 2–3 years [12]. Different thermal expansion coefficients between the core and the veneer, defects in the veneering, and the core wettability could all contribute to this flaw [13].

Monolithic zirconia-based ceramics are an appropriate solution to restore posterior teeth to overcome the clinical complications associated with veneered zirconia. These restorations are produced using CAD/CAM processes and, thus, require fewer steps and less time [14–16]. They can be used in high-load situations, such as for restoring posterior teeth, replacing missing teeth with fixed partial dentures, and managing individuals with heavy masticatory forces and parafunctional habits [17, 18]. Zirconia ceramic has exceptional mechanical properties, namely prevention of crack propagation, low modulus of elasticity, and low thermal conductivity. It is known for its high biocompatibility and low affinity to plaque [4, 19–22]. Moreover, monolithic zirconia crowns require less tooth preparation with reductions of 0.5 mm compared to 1.2–1.5 mm for veneered zirconia crowns [22].

Several variables may reduce the longevity of monolithic zirconia restorations, such as improper crown design, poor tooth preparation, unbalanced load orientation, fabrication techniques, and excessive marginal discrepancy. The cause of failure is typically multifactorial and difficult to determine [23–25]. Thus, the long-term success of fixed dental prostheses highly depends on their marginal integrity. In evaluating monolithic zirconia restorations, precision in their marginal adaptation is critical in determining quality and clinical success. The clinically acceptable marginal discrepancy recommended is less than 120 mm (most commonly between 100 and 120 mm) [26]. Microleakage due to excessive marginal discrepancy could lead to several setbacks that will predispose crowned teeth to secondary caries, increased plaque accumulation, and, eventually, inflammation of the pulp and periodontium [27–29].

The marginal adaptation of a monolithic zirconia crown could be affected during any of the several steps in the CAD–CAM production process. Intraoral scanner precision, CAD software coherence, CAM fabrication procedures optimization, and dental technicians' postmilling adjustments are essential for setting the prosthesis marginal adaptation [7, 30, 31]. Various parameters, including the cement space, can be controlled and modified during the virtual design of such restorations using CAD–CAM technology, which might affect the marginal integrity [32, 33].

Several studies have examined the effect of the cement space on the precision of zirconia crown marginal adaptation, but the results have been contradictory. One study found that different cement spaces (10, 30, and 60 *μ*m) had no substantial influence on the marginal adaptation of the posterior zirconia restorations [34, 35]. Other studies showed that increasing cement thickness improved the marginal adaptation of the crown restoration [25, 36]. However, an internal space larger than 120 mm may have detrimental effects on the prosthesis fracture resistance while not significantly improving the marginal adaptation [31]. Moreover, previous marginal discrepancy studies were more focused on evaluating the vertical marginal gap of different types of fixed prostheses, since the vertical marginal gap is the most difficult to correct after fabrication [35, 37, 38]. Therefore, this study aimed to measure the vertical marginal discrepancy of different cement thickness settings for CAD–CAM monolithic zirconia restorations. The hypothesis is that different cement thickness settings significantly affect the vertical marginal fit of CAD–CAM monolithic zirconia restorations.

## 2. Materials and Methods

The sample was divided into three groups: Group 1, had 10 crowns designed with a 30-*μ*m spacer thickness (the teeth were coded from 0.3 to 9.3); Group 2, had 10 crowns designed with a 50-*μ*m spacer thickness (the teeth were coded from 0.5 to 9.5); and Group 3, had 10 crowns designed with a 70-*μ*m spacer thickness (the teeth were coded from 0.7 to 9.7) [38, 39].

**Figure 1:**
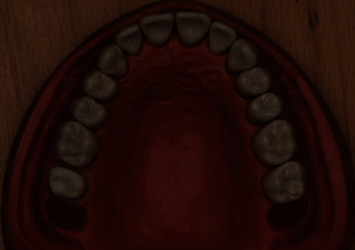
Tooth #17 on a typodont model prepared for a zirconia crown using the standard preparation method.

Before preparation, a putty index was used to achieve greater accuracy. The index was made via the conventional method that uses the condensation silicone putty material (Express STD, 3M ESPE, Seefeld, Germany, Batch no. NA77786). The catalyst and the base were manually mixed at a 1 : 1 ratio until a uniformly colored mass was obtained. An artificial maxillary right molar tooth (#17) was mounted on a typodont model (Frasaco An-4 Puk, Pok) and prepared using round-end tapered diamond bur following the recommended guidelines: occusal reduction 1.5 mm, axial reduction (buccal, palatal, medial, and distal) 1.2–1.4 mm with 2 mm reduction preparation of the functional cusps, rounded internal line angle, tapered walls with 10°–20° of total occlusal convergence, and uniform 1 mm wide rounded shoulder finish line (Figure 1). The prepared tooth was scanned 30 times after it was cleaned and dried using a dental laboratory CAD/CAM scanner (i3Dscan, imes-icore, Eiterfeld, Germany).

**Figure 2:**
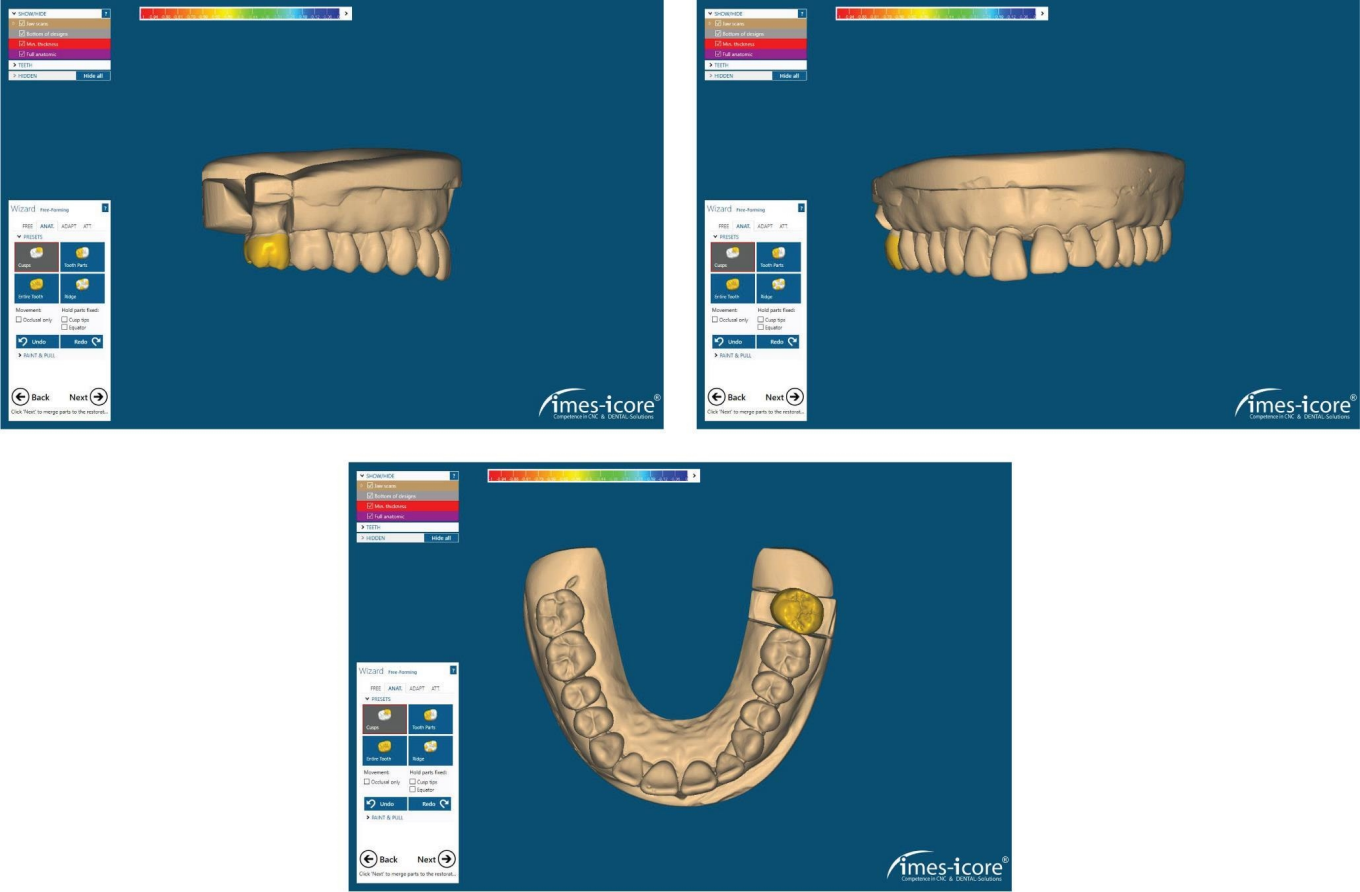
Designing the zirconia crown.

All 30 scans were saved as standard tessellation language files and imported into CORiTEC design software (CORiTEC SmartControl, imes-icore, Eiterfeld, Germany) to be designed following the manufacture instruction for zirconia crown fabrication (Figure 2). In the design phase, the vertical marginal space was set at 0 *μ*m and the cement space was set at 25 *μ*m around the margins for all groups. The files then were randomly distributed with an additional cement space starting 1 mm above the finish lines in 10 crowns in Group 1 with a 30-*μ*m spacer thickness; in the 10 crowns in Group 2 with a 50-*μ*m spacer thickness; and in the 10 crowns in Group 3 with a 70-*μ*m spacer thickness. The Zirconia blocks (Zenostar Zr Translucent Zirconia, Weiland Dental, Pforzheim, Germany) were milled using a CORiTEC 250i Loader Pro system (imes-icore, Eiterfeld, Germany) then fired following the manufacturer's instruction in a Zercomat furnace (VITA Zyrcomat T, VITA Zahnfabrik H. Rauter GmbH & Co. KG).

**Figure 3:**
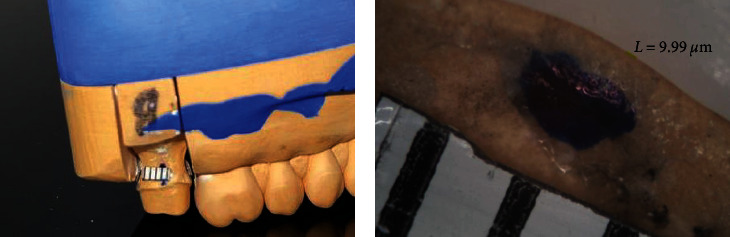
(a) Calibration of the measurements using a ruler on the die. (b) View under the stereomicroscope of the space at the midpoint of the distal margin for Crown #17.

The crowns were numbered, and each crown from each group was seated on the die to evaluate its vertical marginal discrepancy. Neither cement medium nor any other material was used to seat the crowns on the prepared tooth. The vertical marginal discrepancy was measured from four points at the center of each axial surface (the mesial, distal, buccal, and palatal) under a stereomicroscope, which is a reliable and accurate tool for assessing the cement thickness at the margins. The measurements were taken at the middle of each aspect under 40x magnification. A ruler was attached to the die to facilitate the calibration of the measurements on the stereomicroscope (Figures 3(a) and 3(b)). To standardize the assessments of all the locations and groups, only one operator performed all of them. Statistical analysis was conducted using a one-way analysis of variance (ANOVA) test.

## 3. Results

The mean vertical marginal discrepancy of Group 1 (cement space 30 *μ*m) was 27.45, and its standard deviation was 19.84. The mean vertical marginal discrepancy of group 2 (cement space 50 *μ*m) was 22.22, and its standard deviation was 17.19. The mean vertical marginal discrepancy of group 3 (cement space 70 *μ*m) was 22.90, and its standard deviation was 12.82 (Table 1).

The results of the one-way ANOVA test showed that different cement space values did not significantly affect the vertical marginal discrepancy values of the tested crowns (*p* > 0.05). None of the vertical marginal discrepancy locations in all three groups was over the clinically acceptable levels (100–120 *μ*m)

## 4. Discussion

Ensuring vertical marginal adaptation of monolithic zirconia crowns is critical for the longevity of these restorations. In this study, the vertical marginal adaptation of CAD/CAM monolithic zirconia crowns with different cement space settings (30, 50, and 70 *μ*m) did not significantly affect the vertical marginal adaptation of the restorations. Therefore, the null hypothesis was rejected. Theoretically, cement space designation is a dilemma. A narrow cement space may achieve a smaller vertical marginal gap of indirect restorations but would provide difficulty to seat in clinical practice. A wider cement space provides more easier seating but might cause microleakage and loss of restoration retention [40–42]. The result of this study was in accordance with a study by Eldamaty et al. [35], who found that there was no difference in the vertical marginal gap when they used different cement thickness values with zirconia crowns. On the contrary, another study by Kale et al. [38] found that increasing the cement space improves the fit and that a cement space of 25 *μ*m at the margin and 50 *μ*m 1 mm above the margin and elsewhere resulted in the smallest gap of 53 *μ*m compared with 68 and 85 *μ*m in the other groups, where vertical marginal cement spaces were 25 *μ*m, and internal cement space was set at 40 and 30 *μ*m 1 mm above the margin, respectively.

The three different cement space settings were chosen based on previous studies [38, 39]. The least acceptable cement space setting among CAD/CAM restoration is 30 *μ*m, while the suggested setting is 50 *μ*m. This was explained by the fact that a minimum of 30 *μ*m is usually needed for the space of cement, to facilitate the distribution of the cement on the axial walls, and allow coping seating without friction, while the remaining 20 *μ*m was for possible deterioration during production [42, 43]. An additional group with 20 *μ*m above the suggested value was added (70 *μ*m) for further investigation. Crowns were neither cemented nor stabilized on their respective dies with any medium; in clinical scenarios, crowns are usually cemented; however, this study did not consider the effect of different luting agents, cementation procedures, or discrepancies in placement on vertical marginal integrity. It intended to see whether the default cement space in the CAD–CAM software was produced accurately in the manufactured copings or not.

In this study, the clinically acceptable limit (120 *μ*m) for vertical marginal discrepancy was not surpassed at any tested point in all three groups [37]. Although there were no statistically significant differences between the three groups, the results of individual vertical marginal discrepancy values confirmed the results of those reported by previous studies that the number of vertical marginal discrepancy locations reduces with increasing the cement space (27.45 *μ*m in Group 30, 23.22 *μ*m in Group of 50, and 22.9 *μ*m in Group of 70). A narrow cement space value usually prevents the restoration from complete seating, thus increasing the vertical marginal gap. A larger cement space value is beneficial as it would improve the vertical marginal adaptation and reduce the need for internal adjustments. However, the vertical marginal gap should not exceed the recommended values (120 *μ*m) to ensure a long-term success of the restoration. The resultant standard deviation can be attributed to errors in the production, milling, or crystallization, of the zirconia copings, errors in reading of the measurements, and possible seating inaccuracy.

There are few studies in the literature that have assessed vertical marginal discrepancy with different cement thickness values, and further research is needed to evaluate the impact of different cement spaces on the vertical marginal discrepancy using different prosthetic materials and different luting agents. However, this experiment is an in vitro study neither including patient-related variables such as saliva or blood contamination, clinical variables such as preparation designs, luting agents, and seating strategies nor technical variables such as scanning techniques, restoration designing, and production software and machines. Further studies are required to provide guidelines for virtual cement space settings for the various CAD–CAM systems and their impact on the vertical marginal adaptation of different prosthetic restorations.

## 5. Conclusions

Despite the limitations of this in vitro study, we were able to conclude the following:

1. Increasing the cement space from 30 to 50 *μ*m and 70 *μ*m did not significantly impact the vertical marginal adaptation of the CAD–CAM-fabricated monolithic zirconia crowns.
2. Increasing the cement space from 30 to 50 *μ*m and 70 *μ*m did not surpass the clinically acceptable limit of vertical marginal gap value (120 *μ*m).

## Figures and Tables

**Table 1:** Mean and standard deviation of the three different groups.

	Statistic	Standard error
Group 30		
Mean	27.45	3.14
95% confidence interval (CI) for the mean		
Lower bound	21.11	
Upper bound	33.8	
Significance	0.214 > 0.05	
Standard deviation (SD)	19.84	
Minimum	8.81	
Maximum	94.35	
Group 50		
Mean	23.22	2.72
95% confidence interval (CI) for mean		
Lower bound	17.73	
Upper bound	28.72	
Significance		
SD	17.19	
Minimum	5.22	
Maximum	119.73	
Group 70		
Mean	22.9	2.02
95% confidence interval (CI) for mean		
Lower bound	18.79	
Upper bound	27.001	
Significance	0.331 > 0.05	
SD	12.82	
Minimum	9.40	
Maximum	81.96	

## Data Availability

The data presented in this study are available upon request from the corresponding author.

## Additional Points

*Featured Application*. This study proved that different cement spaces do not significantly yield a vertical marginal discrepancy of CAD/CAM monolithic zirconia crown restorations. This allows for greater flexibility in the process of designing these restorations for clinical use.
